# Pseudo-Binary Phase Diagram of LiNH_2_-MH (M = Na, K) Eutectic Mixture

**DOI:** 10.3390/molecules27134093

**Published:** 2022-06-25

**Authors:** Pranjal Pathak, Kriti Shrivastava, Takayuki Ichikawa, Ankur Jain, Rini Singh

**Affiliations:** 1 Centre for Renewable Energy & Storage, Suresh Gyan Vihar University, Jagatpura, Jaipur 302017, India; pranjalpathak7726@gmail.com (P.P.); kriti.shrivastava@mygyanvihar.com (K.S.); 2 School of Applied Sciences, Suresh Gyan Vihar University, Jagatpura, Jaipur 302017, India; 3 Graduate School of Advanced Science & Engineering, Hiroshima University, Higashi-Hiroshima 739-8527, Japan; tichi@hiroshima-u.ac.jp; 4 Natural Science Centre for Basic Research & Development, Hiroshima University, Higashi-Hiroshima 739-8530, Japan

**Keywords:** lithium amide (LiNH_2_), sodium hydride (NaH), potassium hydride (KH), eutectic melting, phase diagram

## Abstract

The hunt for a cleaner energy carrier leads us to consider a source that produces no toxic byproducts. One of the targeted alternatives in this approach is hydrogen energy, which, unfortunately, suffers from a lack of efficient storage media. Solid-state hydrogen absorption systems, such as lithium amide (LiNH_2_) systems, may store up to 6.5 weight percent hydrogen. However, the temperature of hydrogenation and dehydrogenation is too high for practical use. Various molar ratios of LiNH_2_ with sodium hydride (NaH) and potassium hydride (KH) have been explored in this paper. The temperature of hydrogenation for LiNH_2_ combined with KH and NaH was found to be substantially lower than the temperature of individual LiNH_2_. This lower temperature operation of both LiNH_2_-NaH and LiNH_2_-KH systems was investigated in depth, and the eutectic melting phenomenon was observed. Systematic thermal studies of this amide-hydride system in different compositions were carried out, which enabled the plotting of a pseudo-binary phase diagram. The occurrence of eutectic interaction increased atomic mobility, which resulted in the kinetic modification followed by an increase in the reactivity of two materials. For these eutectic compositions, i.e., 0.15LiNH_2_-0.85NaH and 0.25LiNH_2_-0.75KH, the lowest melting temperature was found to be 307 °C and 235 °C, respectively. Morphological studies were used to investigate and present the detailed mechanism linked with this phenomenon.

## 1. Introduction

The steady depletion of fossil fuels and their hazardous byproducts has led to the search for alternative renewable energy sources [1,2]. The availability of renewable energy sources varies depending on weather and location, which has drawn the attention of many researchers and has expedited the development of efficient energy storage and energy transport technologies. With its byproduct, water, hydrogen is one of the most promising energy carriers for automotive applications. [3,4,5]. However, hydrogen storage technology is very challenging for many researchers due to its low density with the known possibilities provided by compressed and liquid hydrogen [6] for several applications. However, when it comes to the use of hydrogen in our daily lives, several issues come up. To address the key issues such as low density and very low temperature of liquid hydrogen, i.e., 20.2 K [7,8], two possible methods are high-pressure tanks and cryogenics, but they are difficult to implement for daily life applications [9,10].

Light element hydrides (LiH, MgH_2_, LiNH_2_, NaAlH_4,_ etc.) are suitable candidates for hydrogen storage in solid form due to their increased gravimetric and volumetric hydrogen densities at normal conditions [11,12,13,14,15]. Several studies of hydride and their combinations for hydrogen storage have been reported [16,17]. Additionally, the combination of solid hydrides (LiH and LiNH_2_), known as an amide-imide (M-N-H) hydrogen system, is also extensively studied [18,19]. Chen et al. published the first report on the LiNH_2_ system, well-known for storing hydrogen up to 10.4 wt% via the following conversion reactions [20]:

Li_3_N + 2H_2_ → Li_2_NH + LiH + H_2_ ↔ LiNH_2_ + 2LiH (1)

Kojima et al. found the desorption enthalpy change as 65.6 kJ mol^−1^ [21] for the same reaction, while Ichikawa et al. reported the reaction through the evolution of ammonia from LiNH_2,_ i.e.,

2LiNH_2_ → Li_2_NH + NH_3_ (2)

LiH+NH_3_ →LiNH_2_ + H_2_ (3)

Hydrogen can be desorbed as per these reactions below the temperature of 300 °C. Several catalysts and additives have been explored to improve the reaction kinetics. In this direction, potassium and sodium hydride has attained significant attention due to their extraordinary performance. The KH addition reduced the hydrogen desorption temperature of the Mg(NH_2_)-LiH system from 186 °C to 107 °C, according to Wang and coworkers. The diffusion of potassium into amide and imide, which, when paired with nitrogen, weakens the N-H and Li-N bonds and so promotes dehydrogenation, has been proposed as the basis for this exceptional performance [22]. Similarly, Teng et al. reported the improved hydrogen desorption kinetics of the LiH-NH_3_ system by adding a small amount of KH. The improved reactivity of KH with NH_3_ (emitted from Mg(NH_2_)_2_), resulting in KNH_2_ and H_2_, was suggested as a probable reason for the improvement. By solid-solid interaction, this in situ produced KNH_2_ reacts with LiH and forms LiNH_2_ and KH as a reaction product [23]. 

The LiNH_2_-KH composite system was explored in our recent work, and the ammonolysis rate was greatly improved. The reaction product was discovered to be a double-cation amide phase LiK(NH_2_)_2_ [24,25]. Because of the eutectic melting phenomena, the reaction temperature was determined to be lower than the individual melting points of LiNH_2_ and KH. Basically, eutectic melting is the lowering of the melting temperature of a mixture of two materials as compared to their respective melting temperatures without any change in their phases. This destabilization of the associated species allows low-temperature hydrogen release and has sparked much interest in hydrogen storage materials, particularly complicated hydrides, melting at lower temperatures. Low-melting-point hydrogen storage materials act like ionic liquids and enable quick vehicle refilling, like present fossil-fuel technologies. Several studies on the eutectic melting of metal borohydrides have been published [26,27]. After the discovery of the eutectic phenomenon for the LiNH_2_-KH system, its detailed investigation has become imperative. In the present study, along with the addition to KH, NaH additive was also considered to visualize and understand the eutectic phenomenon in amide-hydride systems. The thermal studies were performed using the differential scanning calorimetry (DSC). XRD was used to validate the presence of individual phases and their intactness, while the SEM technique was used to investigate the unique characteristic of eutectic melting through morphological characterization. Using DSC thermograms, a complete pseudo-binary phase diagram was produced for a number of compositions with varied atomic ratios.

## 2. Results and Discussion

**Figure 1:**
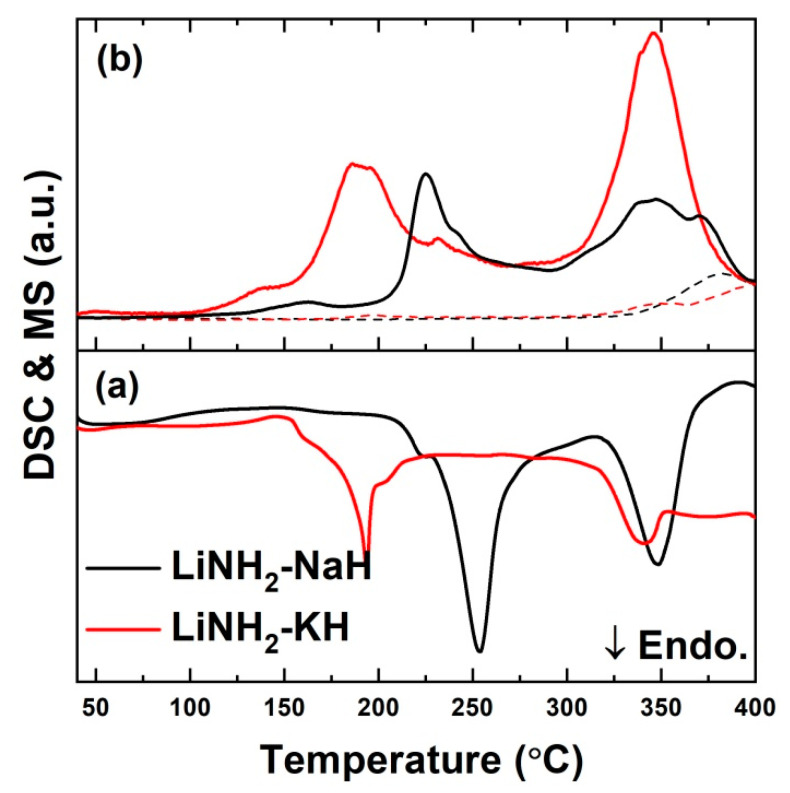
(**a**) DSC profile of LiNH_2_-KH and LiNH_2_-NaH system (**b**) Thermal desorption mass spectroscopy of LiNH_2_-KH and LiNH_2_-NaH system (H_2_ spectrum is shown by solid line and NH_3_ spectrum is shown by dashed line).

DSC and mass spectroscopy were used on these samples to determine the thermal characteristics of LiNH_2_-MH complexes. As shown in Figure 1a, DSC measurements were carried out in a 0.1 MPa Ar environment up to 400 °C at a scan rate of 5 °C/min. In previous research, we looked into the thermal decomposition of the LiNH_2_ -NaH (1:1 ratio) system [28]. This system does not show any sharp/significant peak up to 200 °C, but only a broad exothermic hump was observed, which was suggested due to the ionic mobility of LiNH_2_ and NaH, resulting in the formation of Li_3_Na (NH_2_)_4_ [29]. The above report also clearly stated further disintegration.

In a similar way, the thermal decomposition of the LiNH_2_-KH (1:1 ratio) system has been analyzed in this study. Two endothermic peaks appeared at 193 °C and 347 °C, along with a minor exothermic shoulder peak at 149 °C. It is noteworthy here that only H_2_ evolution occurred for the entire temperature range, as evident from the corresponding MS signals (Figure 1b; solid lines). Only a small amount of NH_3_ (Figure 1b; Dashed lines) was detected at higher temperatures (>350 °C). This exothermic peak at 149 °C can be speculated as to the formation of a double-cation amide phase, comparable to the Li_3_Na(NH_2_)_4_ in the other system. It is noteworthy here that no melting was observed during the thermal heating of both systems under the Ar atmosphere, as evident from the absence of any sharp endothermic peaks in the studied range. It must be due to the transformation of initial species to some complex amides such as Li_3_Na(NH_2_)_4_ and Li_3_K(NH_2_)_4_ during milling/heating at very low temperatures. 

**Figure 2:**
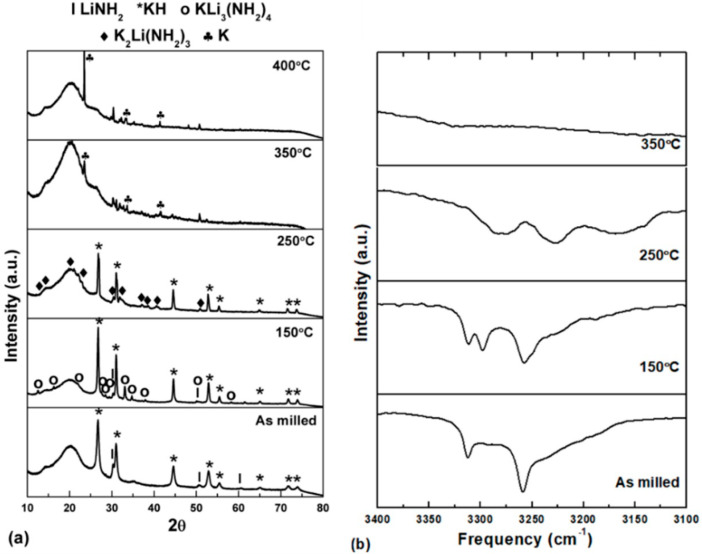
(**a**) XRD pattern and (**b**) FTIR spectra of LiNH_2_ and KH milled sample at different temperatures.

XRD investigations were performed after this temperature, and the results are shown in Figure 2a. The XRD analysis indicates the presence of Li_3_K(NH_2_)_4_ in addition to the initial phases, i.e., LiNH_2_ and KH for the sample heated up to 150 °C. The endothermic peaks at 193 °C and 347 °C are very similar to the reaction of LiNH_2_ and NaH. Heating up to 250 °C transformed the mixture of Li_3_K(NH_2_)_4_, LiNH_2,_ and KH into K_2_Li(NH_2_)_3_ and KH, which is evident from the XRD profile (Figure 2a). Further heating to higher temperatures leads to the decomposition of these amides and the evolution of H_2._ It also shows the final reaction product to be the potassium metal only (top panel of Figure 2a). 

FTIR experiments were conducted at various stages of heating to better explain this phenomenon and establish the presence and transition of amide-imide. Figure 2b shows a summary of the findings. The presence of LiNH_2_ is confirmed by two distinctive peaks in the FTIR spectra of a milled sample at 3312 and 3259 cm^−1^. Furthermore, a distortion in the peak at 3259 cm^−1^ suggests a minor interaction between LiNH_2_ and KH, resulting in the formation of a small percentage of double-cation amide, which was too small to be seen in the XRD profile. Heating the sample up to 150 °C transformed it into double-cation amide along with the starting materials. This is supported by the FTIR spectra, which show an additional set of peaks. Since the new peaks are shifted toward the lower-frequency side (red shifted as compared to the peak of LiNH_2_), these can be considered as K-substituted LiNH_2_ structure, i.e., Li_3_K(NH_2_)_4_. A clear peak at 3298 cm^−1^ and overlapped peak at 3253 cm^−1^ (Figure 2b) are in suitable agreement with the observations of Dong et al. [29], where they reported Li_3_K(NH_2_)_4_ as an important intermediate compound. 

Further heating to a higher temperature, i.e., 250 °C, increases the reactivity between KH, LiNH_2,_ and previously formed Li_3_K(NH_2_)_4_, which turns them into another double-cation phase K_2_Li(NH_2_)_3_ with higher K content. This can again be seen in the FTIR spectra, where a red shift in the peaks is observed as compared to that of the Li_3_K(NH_2_)_4,_ and the new peaks are developed at 3286 and 3227 cm^−1^. Additionally, a broad characteristic peak around 3160 cm^−1^ corresponds to the existence of Li_2_NH. However, the presence of Li_2_NH could not be confirmed through XRD, indicating the presence of amorphous characteristics. Further heating up to 350 °C causes the disappearance of the peaks in the FTIR spectra, suggesting the decomposition of the amide-imide phase into a metallic state, also evidenced in the XRD profile.

**Figure 3:**
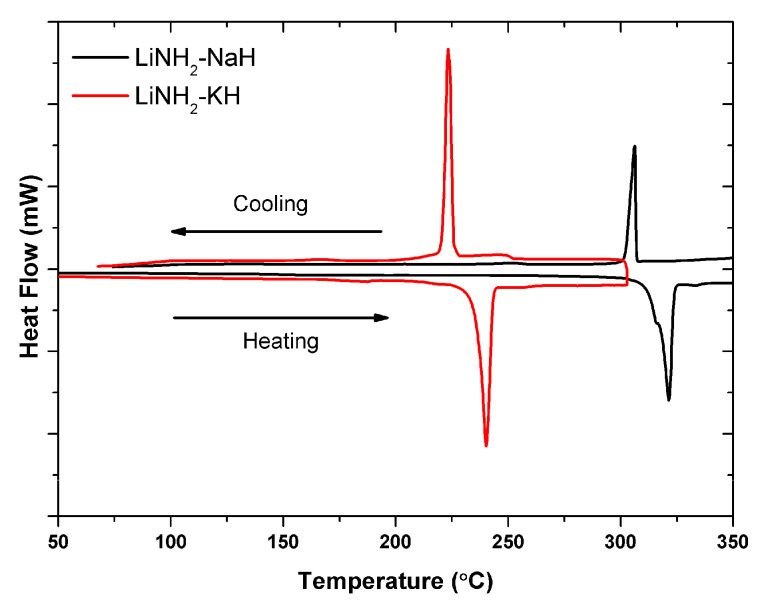
DSC thermogram of 0.5LiNH_2_-0.5NaH and 0.5LiNH_2_-0.5KH composite samples. The DSC measurements were performed under the closed conditions under a 0.5 MPa H_2_ atmosphere up to 370 °C with a heating rate of 5 °C/min.

The reaction atmosphere has a significant impact on the thermal behavior of hydrogen storage materials [30,31]. The existence of gas species in the reaction field can have a considerable impact on the reaction mechanism of such a complex system. It is due to the fact that the presence of Ar works as a vacuum condition where the thermodynamics can not be changed; however, the presence of H_2_ in the reaction field creates a back pressure of hydrogen toward the reaction and affect the thermodynamic significantly due to which the reaction pathways can be altered drastically. Generally, it is seen that the presence of hydrogen in the reaction field shifts the decomposition temperature to the higher side [31]. In contrast to the decomposition under the Ar atmosphere reported in this work, it was observed in our previous studies that the LiNH_2_-KH system did not undergo decomposition even under a small H_2_ pressure of 0.5 MPa, and this system revealed the possibility of eutectic melting. To understand the mechanism and its relevance to other systems, detailed investigations were performed on LiNH_2_-MH (M = Na, K) systems under 0.5 MPa H_2_. Figure 3 shows the DSC thermograms of both samples during heating and cooling cycles. 

For the LiNH_2_-KH sample, a reversible peak is obtained at around 240 °C, whereas the melting temperatures of individual LiNH_2_ and KH are 390 °C and 400 °C. Furthermore, it is important to note that the temperatures of these melting and solidification peaks did not change with varying hydrogen pressures (not shown here). Due to the existence of relatively identical peak positions, any disintegration during heating could be ruled out (the origin of the cooling peak coincides with the origin of the heating peak). The LiNH_2_-NaH sample showed a similar set of peaks but at a higher temperature, 321 °C, which is lower than the individual melting temperatures of LiNH_2_ and NaH. These DSC results point to eutectic melting as a possibility. Furthermore, the physical appearance of both samples after the above-mentioned DSC experiments indicated the sign of melting.

**Figure 4:**
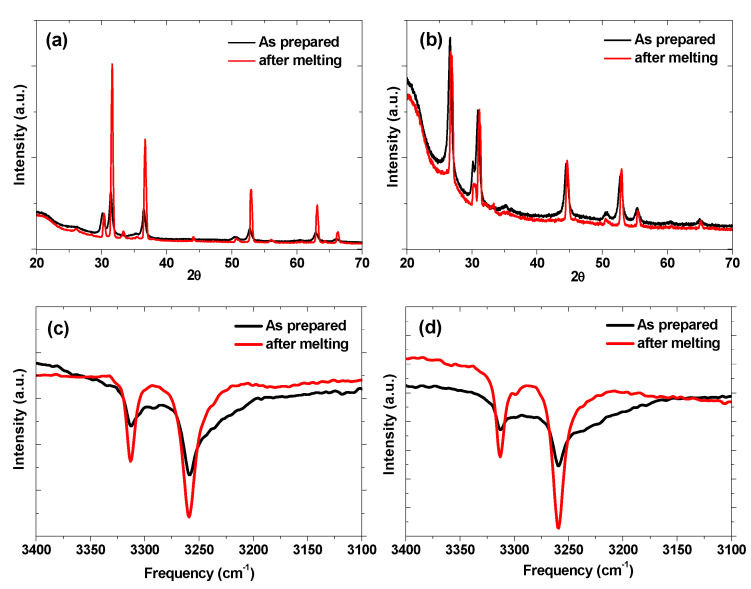
(**a**) XRD pattern of LiNH_2_-KH sample before and after DSC measurement. (**b**) XRD pattern of LiNH_2_-NaH; (**c**) FTIR spectra of LiNH_2_-KH sample; (**d**) FTIR spectra of LiNH_2_-KH sample.

XRD was performed on both the samples after melting to obtain scientific evidence of this low-temperature eutectic melting. The XRD and FTIR profiles of both the samples before and after melting (Figure 4a,b) can be used to establish the eutectic melting phenomenon as they reflect the intact nature of participating components. They suggest the improvement in crystallinity after melting without changing the existing phases or introducing the new phases. Additionally, the FTIR spectra of both the samples (Figure 4c,d) also do not show any new peak after melting. In fact, the observed distortion in the low-frequency peak for both as prepared samples disappeared after melting. This suggests the dissolution of a small fraction of double-cation amide under the hydrogen atmosphere during melting. These findings support the hypothesis of eutectic melting in these systems. 

**Figure 5:**
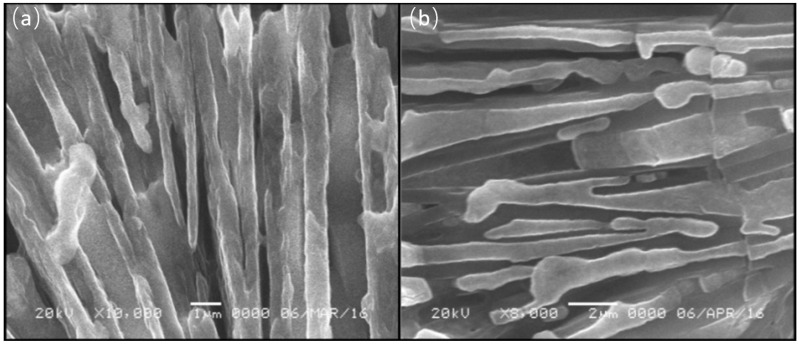
Scanning electron micrographs (**a**) 0.5LiNH_2_- 0.5NaH system (**b**) 0.5LiNH_2_ –0.5KH systems.

According to the basic definition of eutectic melting, atom diffusion into the other phase element or preferential segregation of the two-phase elements can result in the production of unique microstructures. The samples were subjected to scanning electron microscopy to validate this. Figure 5 shows the SEM results, which demonstrate the presence of distinctive lamellar microstructures in both samples. This indicates that the growth is coupled, implying that the phases of LiNH_2_, NaH, and KH form a liquid at the same interface, whether it is isothermal or planar. Rutter and coworkers reported this phenomenon occurring at the interface of all the phases [32]. The large-sized grains observed in these samples can be attributed to the perfect lamellar structure [30,31,32]. Efforts have been made by researchers to find out the different microstructures of eutectics. According to Chadwick et al., the growth of lamellar eutectics alloys is caused by the simultaneous edgewise growth of phases of both materials in the presence of constant heat flow [33]. The confirmation of eutectic melting through all of the foregoing evidence prompted the investigation of the exact eutectic composition of the LiNH_2_-NaH and LiNH_2_-KH systems, which have the lowest eutectic temperature that might be used in future research.

**Figure 6:**
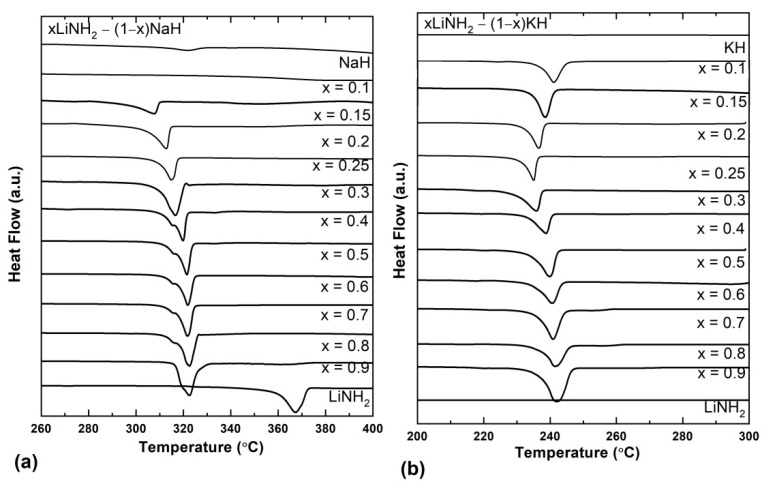
(**a**) DSC thermograms of xLiNH_2_-(1−x) NaH (**b**) DSC thermograms of xLiNH_2_-(1−x) KH at different compositions.

Detailed investigation of the composition and temperature of eutectic melting was carried out using DSC thermograms taking different compositions of xLiNH_2_-(1−x)NaH and xLiNH_2_-(1−x)KH systems where x = 0, 0.1, 0.15, 0.2, 0.25, 0.3, 0.4, 0.5, 0.6, 0.7, 0.8, 0.9, and 1 for both systems. These DSC curves under 0.5 MPa H_2_ are shown in Figure 6. Let’s first discuss the xLiNH_2_-(1−x) NaH compositions shown in Figure 6a. 

The melting temperature of individual LiNH_2_ and NaH is 368 °C and >800 °C, respectively. The DSC thermogram shows the absence of any melting peak for NaH in the temperature range of 200–300 °C, which remained the same for the composition 0.1LiNH_2_-0.9NaH. When the LiNH_2_ content was further increased, i.e., 0.15LiNH_2_-0.85NaH, an endothermic peak corresponding to the melting appeared at 307 °C and increased continuously with the increase in the content of LiNH_2_. Similar DSC experiments were performed for the xLiNH_2_-(1−x) KH compositions, and the results are depicted in Figure 6b. 

The individual melting point of LiNH_2_ and KH is 368 °C and 400 °C, respectively. It is observed that the bare samples of LiNH_2_ and KH show no melting peaks in the given temperature range. For the composition 0.1LiNH_2_ –0.9 KH, an endothermic melting peak was observed at 240 °C, and on further increasing the LiNH_2_ content, the melting temperature is decreased. The lowest melting temperature was observed at 235 °C for the 0.25LiNH_2_ –0.75 KH composition, which is much lower than that of the individual melting points of the LiNH_2_ and KH. XRD of all the studied xLiNH_2_-(1−x)NaH and xLiNH_2_-(1−x)KH samples suggest no change in the initial phases except for the formation of better crystallinity after melting (Figures S1 and S2). A similar observation was received from FTIR spectra. The morphological investigation of the series of these samples (Figures S3 and S4) clearly visualizes the melting phenomena, thus ruling out any possibility of desorption corresponding to DSC endothermic peaks (Figure 6).

**Figure 7:**
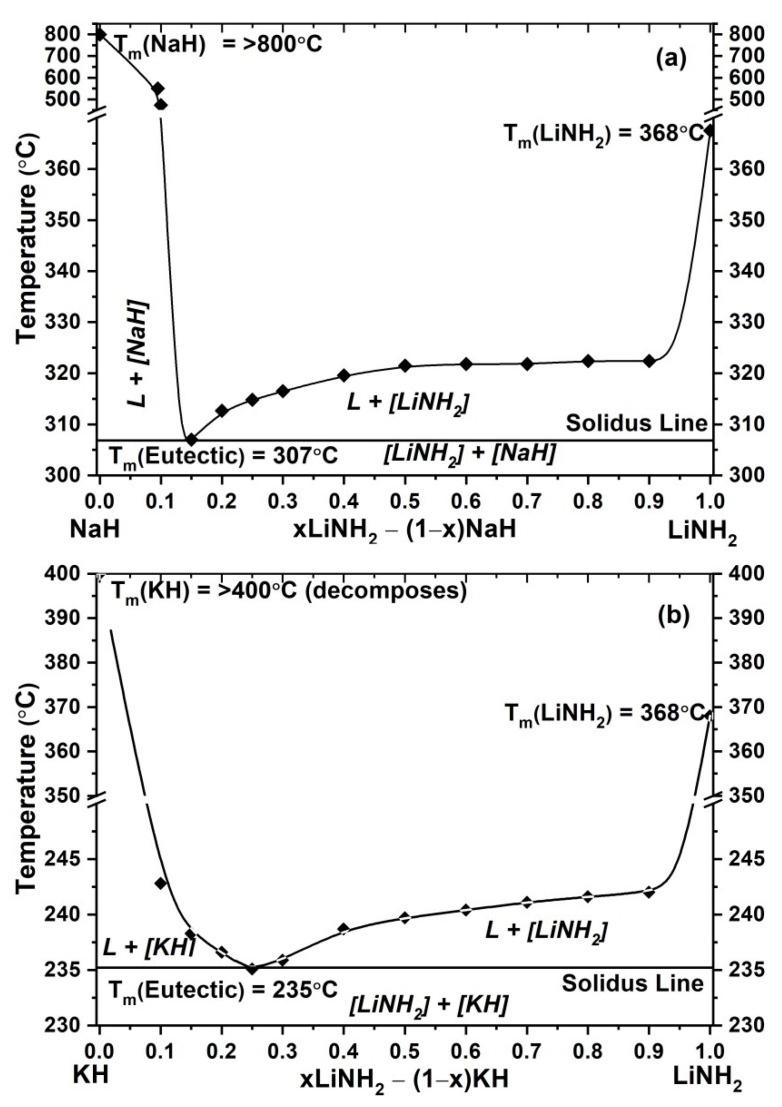
Phase diagram of LiNH_2_-NaH (**a**) and LiNH_2_-KH (**b**) system. Consideration of onset temperature as melting point has been taken. (L represents the melted state in the figure).

The melting temperatures of each individual composition were determined using the above DSC data, and a phase diagram of these systems was prepared. The pseudo-binary phase diagram for xLiNH_2_-(1−x) NaH is shown in Figure 7a, which clearly shows a reduction in the melting temperature of NaH from more than 800 °C to 320 °C when a small amount of LiNH_2_ is mixed in NaH. The melting temperature of NaH is 800 °C, with the addition of LiNH_2_, the temperature is reduced systematically while in the composition 0.15LiNH_2_ –0.85NaH the eutectic temperature was found to be 307 °C. The confirmation of eutectic composition can be done on the basis of the similar crystal structure and observed morphology. Similarly, for the composition xLiNH_2_-(1−x) KH, Figure 7b suggested the eutectic composition is 0.25LiNH_2_ –0.75KH with the eutectic temperature of 242 °C. 

## 3. Materials and Methods

### 3.1. Sample Preparation

LiNH_2_ (95%), NaH (95%), and KH (95%) were purchased from Sigma Aldrich and processed further. The LiNH_2_ and MH (M = Na, K) were milled in different molar ratios from 0.1 to 1 with a step of 0.1 to prepare 11 different compositions (including 2 samples of pure LiNH_2_ and MH). The ball milling (Fritsch P7 ball milling apparatus) was the preferred technique for sample preparation. A total of 20 Cr steel balls (SUJ- 2 having 7 mm diameter) were used for 1 g sample batch. The milling was performed for a total of 2 h with 1 h milling and 30 min rest pattern at the initial Ar pressure of 0.1 MPa and rotation speed of 400 rpm. Due to the high sensitivity of the materials, all the samples were handled in the Argon atmosphere with oxygen, and moisture content was maintained below 1 ppm using a high-purity Ar-filled glove box (Miwa MFG, MP-P60W).

### 3.2. Characterization of Samples

Structural studies of the samples prepared before and after melting were carried out by X-ray diffraction using Rigaku RINT 2500 equipped with CuKα radiation. To avoid interaction of the samples to air, samples were covered with polyamide sheets and vacuum grease. For bonding identification, Fourier transforms Infrared spectroscopy (FTIR Perkin Elmer spectrum) was used. The thermal properties of the synthesized samples were explored using differential scanning calorimetry (DSC, TA Instruments Q10PDSC). The DSC measurements on all the samples were performed under two different atmospheres, i.e., under 0.1 MPa Ar and 0.5 MPa H_2_ atmospheres. The identification of gaseous species during the thermal heating under a 0.1 MPa Ar atmosphere was investigated by thermal desorption mass spectroscopy (TDMS) (Canon Anelva Corporation, M-100QA) (Kawasaki, Japan). The morphological studies of each sample were performed by scanning electron microscopy (SEM) using a JEOL, JSM-6380A instrument. SEM samples were also prepared in the glove box to avoid air exposure. 

## 4. Summary

The reduction in melting temperature caused by the addition of KH and NaH to the LiNH_2_ system is investigated. The reaction mechanism varies depending on the reaction field’s atmosphere. Both systems decompose when heated to 0.1 MPa; however, the presence of 0.5 MPa H_2_ prevents the decomposition altogether. In addition, the presence of H_2_ in the reaction field resulted in an unexpected eutectic melting phenomenon. With the use of DSC thermograms, we were able to build a pseudo-binary phase diagram for these systems after further analysis. The eutectic temperature for the eutectic composition 0.15LiNH_2_-0.85NaH was found to be 307 °C, but the lowest melting temperature for the xLiNH_2_-(1−x)KH system was found to be 235 °C for x = 0.25.

## Data Availability

Data available from authors.

## Supplementary Materials

The following supporting information can be downloaded at https://www.mdpi.com/article/10.3390/molecules27134093/s1, Figure S1: XRD (left) and FTIR (right) spectra of xLiNH_2_-(1−x)NaH before and after melting; Figure S2: XRD (left) and FTIR (right) spectra of xLiNH_2_-(1−x)KH before and after melting; Figure S3: SEM of xLiNH_2_-(1−x)NaH before and after melting; Figure S4: SEM of xLiNH_2_-(1−x)KH before and after melting.

## Sample Availability

Samples are available from the authors.
