# Supplementary material for: Pseudo-Binary Phase Diagram of LiNH2-MH (M = Na, K) Eutectic Mixture
Source: Molecules. 2022 Jun 25;27(13):4093. doi: 10.3390/molecules27134093 (PMC9268627; doi:10.3390/molecules27134093)
Supplement: Supplementary file 1 [file molecules-27-04093-s001.zip › molecules-1779243-supplementary.pdf]

Supplementary Material

Pseudo-Binary Phase diagram of $LiNH_2$-MH (M = Na, K) Eutectic Mixture

Pranjal Pathak [1], Kriti Shrivastava [1,2], Takayuki Ichikawa [3], Ankur Jain [1,4,*] and Rini Singh [3,*]

1. Centre for Renewable Energy & Storage, Suresh Gyan Vihar University, Jagatpura, Jaipur 302017, India; pranjalpathak7726@gmail.com (P.P.); kriti.shrivastava@mygyanvihar.com (K.S.)
2. School of Applied Sciences, Suresh Gyan Vihar University, Jagatpura, Jaipur 302017, India
3. Graduate School of Advanced Science & Engineering, Hiroshima University, Higashi-Hiroshima 739-8527, Japan; tichi@hiroshima-u.ac.jp
4. Natural Science Centre for Basic Research & Development, Hiroshima University, Higashi-Hiroshima 739-8530, Japan

* Correspondence: ankur.j.ankur@gmail.com (A.J.); rini@hiroshima-u.ac.jp (R.S.)

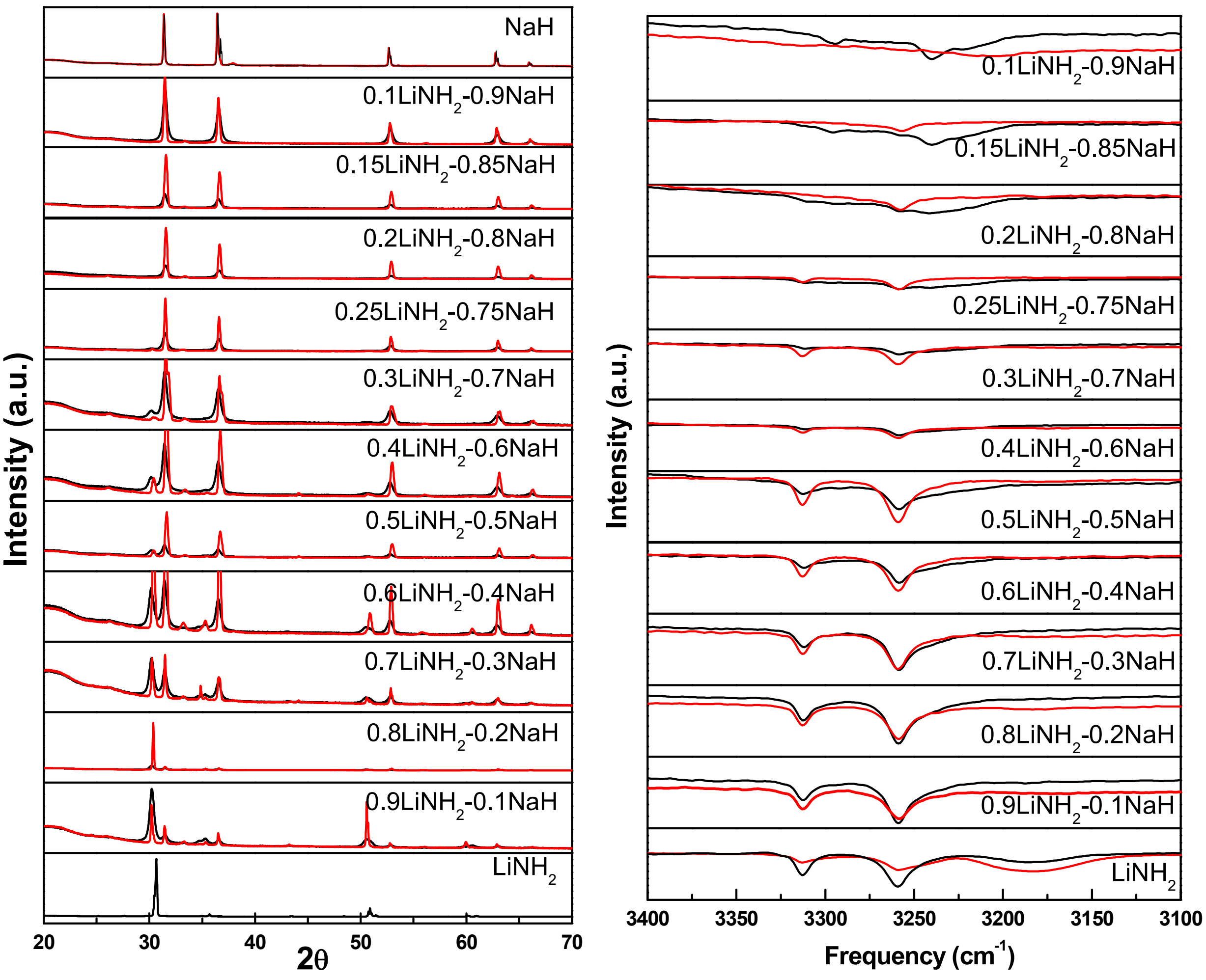

Figure S1. XRD (left) and FTIR (right) spectra of $xLiNH_2$ - (1-x)NaH before and after melting.

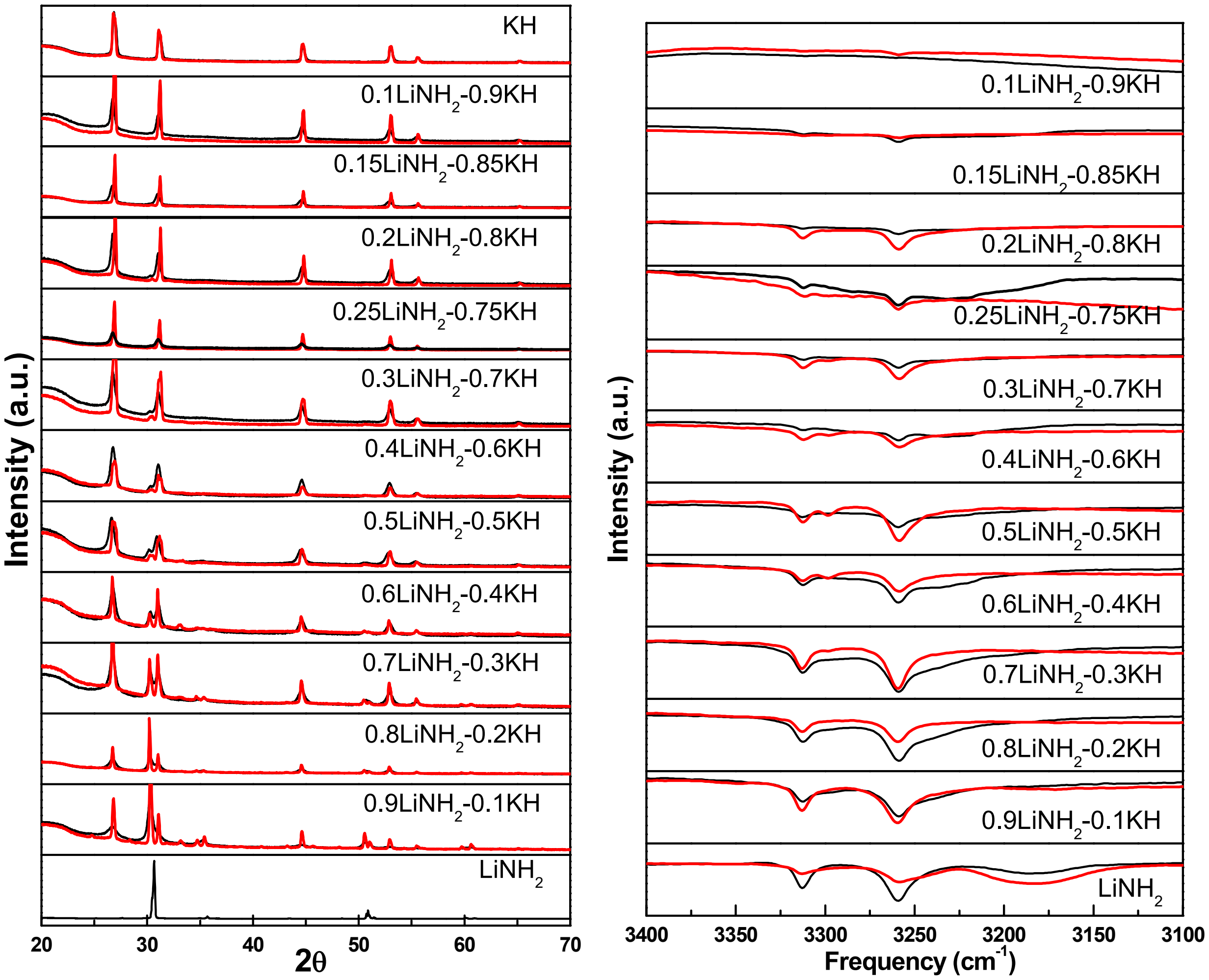

Figure S2. XRD (left) and FTIR (right) spectra of xLiNH2 - (1-x)KH before and after melting.

Pseudo-Binary Alloy System	As prepared	After Melting
$LiNH_2$	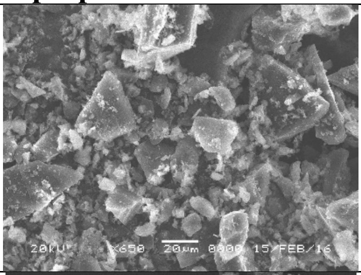	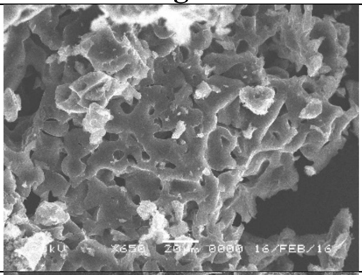
$LiNH_2$-0.1NaH	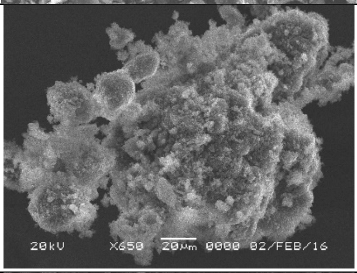	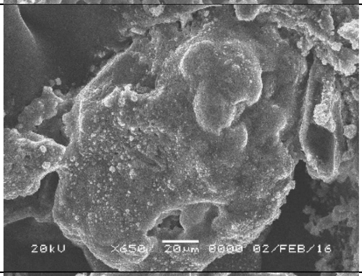
$LiNH_2$-0.2NaH	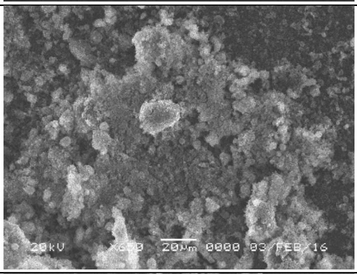	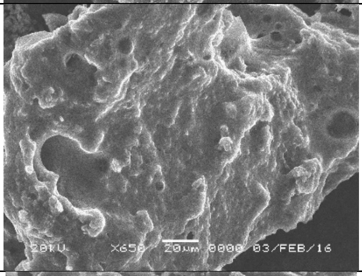
$LiNH_2$-0.3NaH	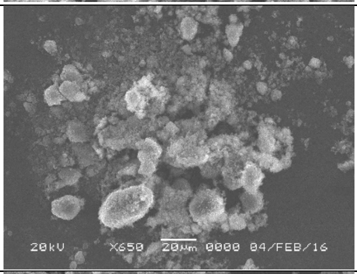	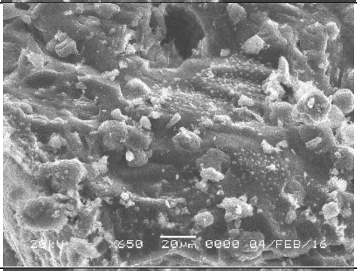
$LiNH_2$-0.4NaH	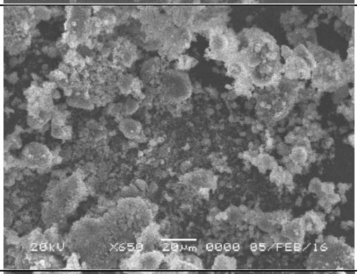	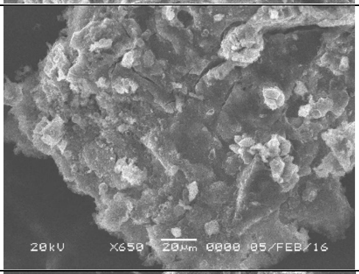
$LiNH_2$-0.5NaH	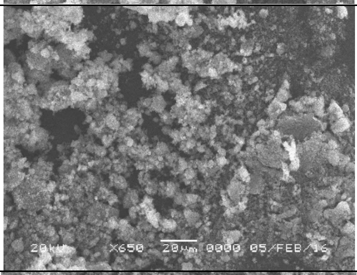	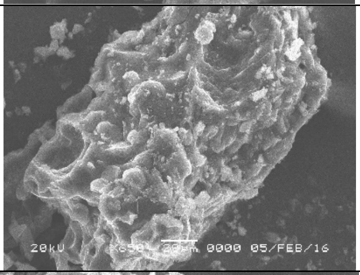
$LiNH_2$-0.6NaH	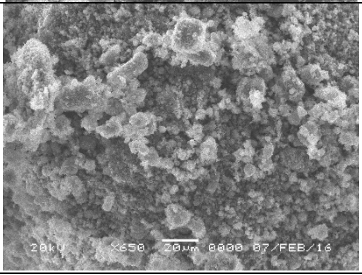	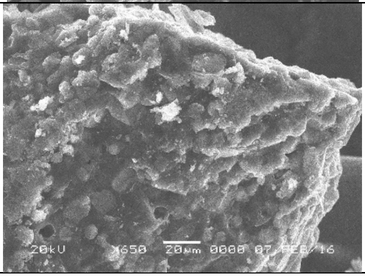

**$LiNH_2$-0.7NaH**	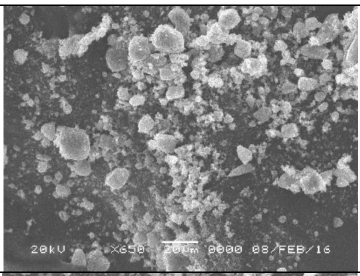	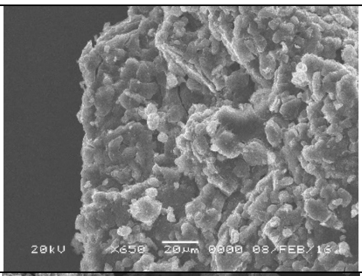
**$LiNH_2$-0.75NaH**	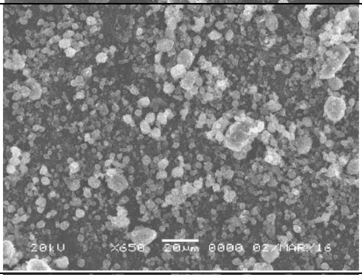	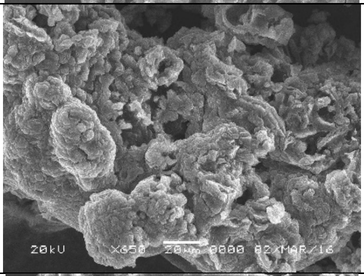
**$LiNH_2$-0.8NaH**	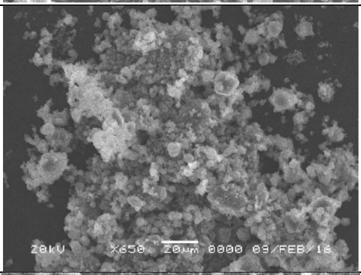	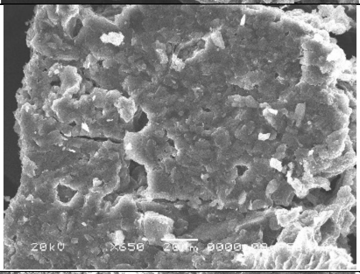
**$LiNH_2$-0.85NaH**	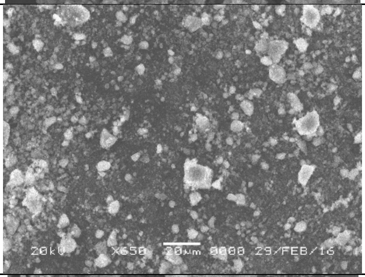	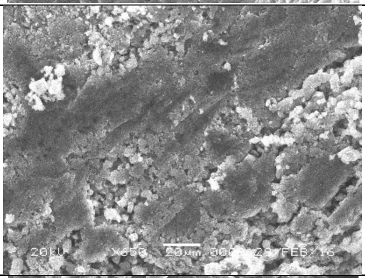
**$LiNH_2$-0.9NaH**	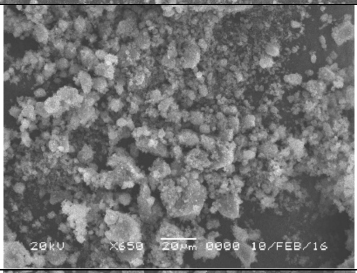	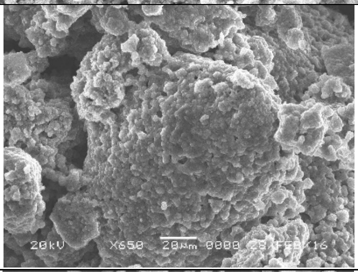
**NaH**	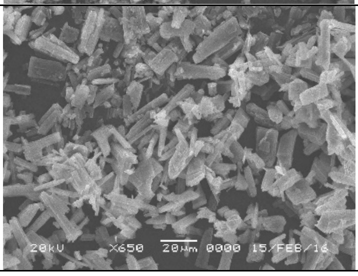	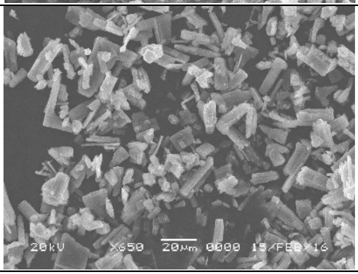

Figure S3. SEM of $xLiNH_2$ - (1-x)NaH before and after melting.

Pseudo-Binary Alloy System	As prepared	After Melting
$LiNH_2$	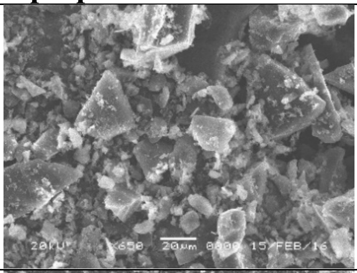	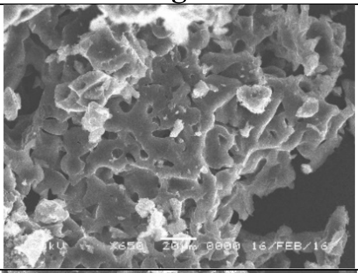
$LiNH_2$-0.1KH	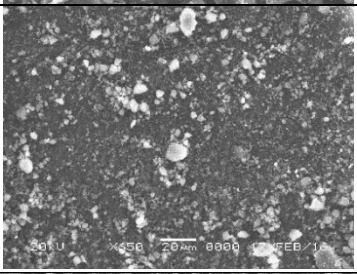	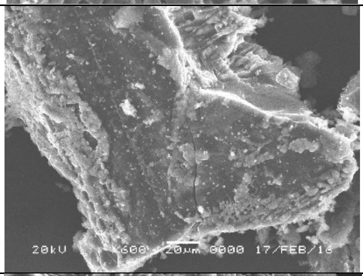
$LiNH_2$-0.2KH	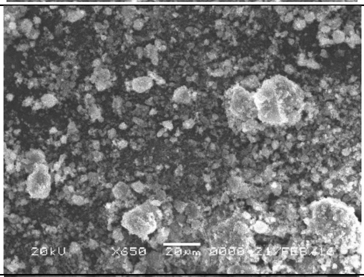	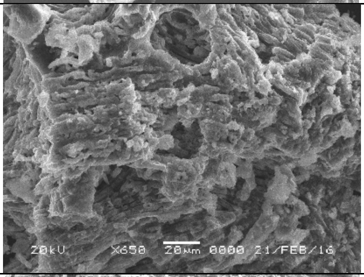
$LiNH_2$-0.3KH	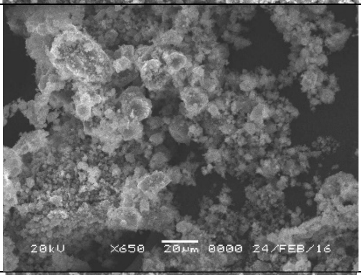	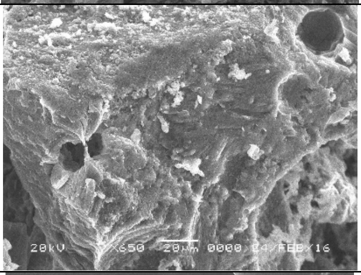
$LiNH_2$-0.4KH	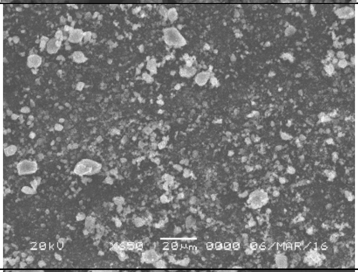	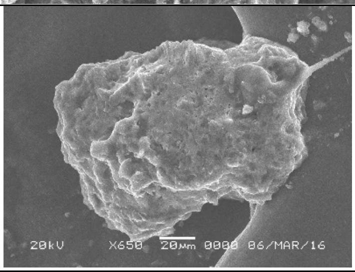
$LiNH_2$-0.5KH	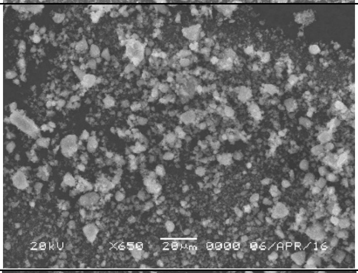	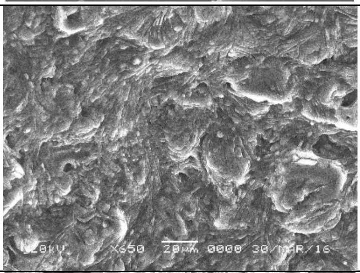
$LiNH_2$-0.6KH	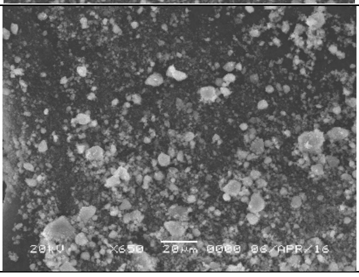	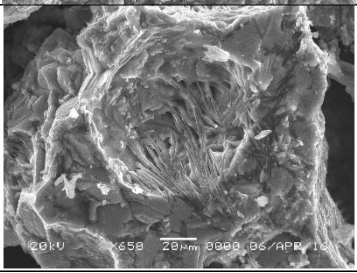

**LiNH$_2$-0.7KH**	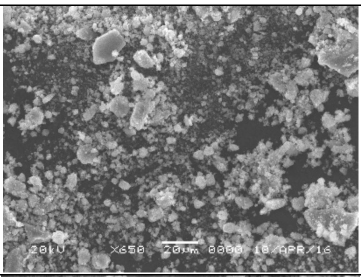	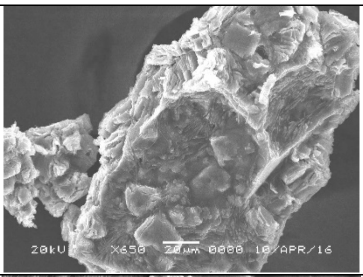
**LiNH$_2$-0.75KH**	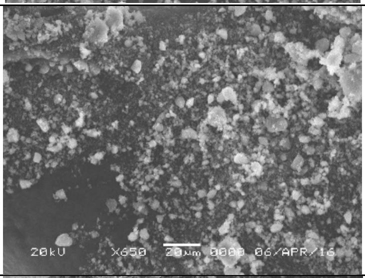	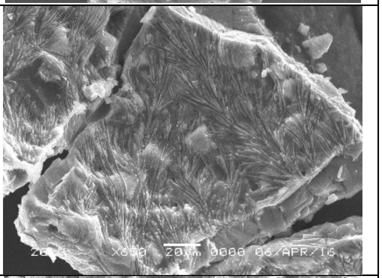
**LiNH$_2$-0.8KH**	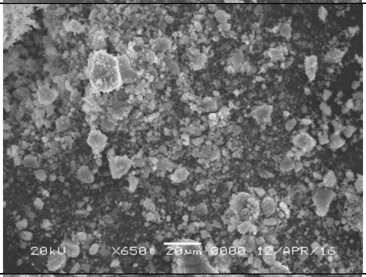	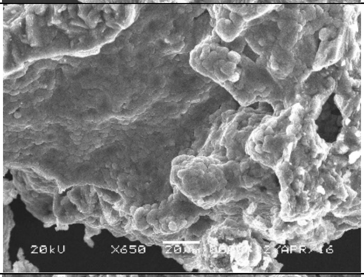
**LiNH$_2$-0.85KH**	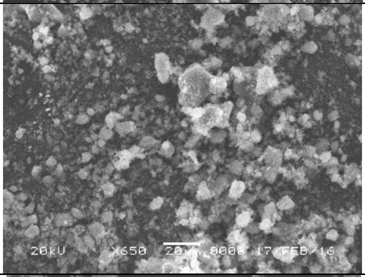	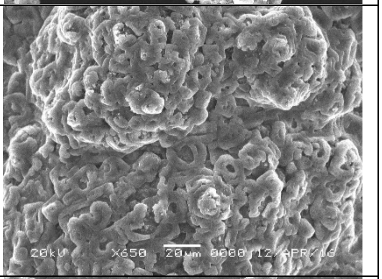
**LiNH$_2$-0.9KH**	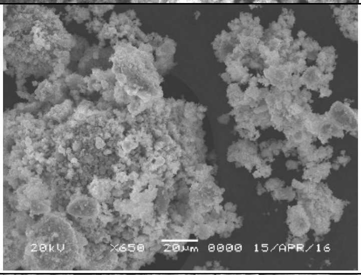	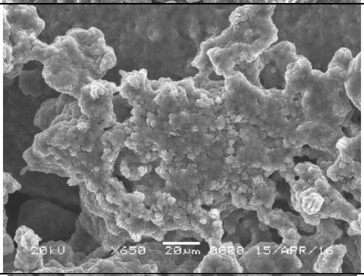
**KH**	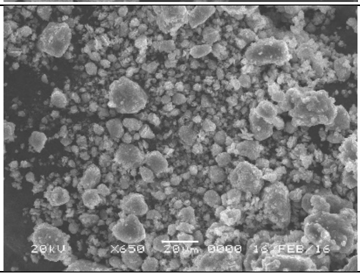	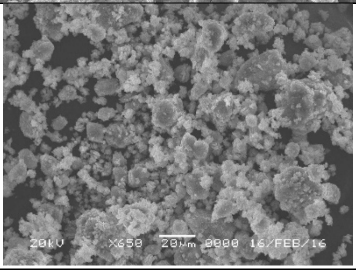

Figure S4. SEM of xLiNH$_2$ - (1-x)KH before and after melting.
